# It takes a village: A pilot cross-randomized trial to enhance pregnancy care and support in northern Ghana

**DOI:** 10.7189/jogh.14.04217

**Published:** 2024-10-25

**Authors:** Aleksandra Jakubowski, Raymond Akawire Aborigo, Irene Kuwolamo, Jesse D Meredith, Aaron Asibi Abuosi

**Affiliations:** 1 Department of Public Health and Health Sciences, Department of Economics, Northeastern University, Boston, Massachusetts, USA; 2 Navrongo Health Research Centre, Navrongo, Ghana; 3 Department of Economics, Boston University, Boston, Massachusetts, USA; 4 Department of Health Policy, University of Ghana, Accra, Ghana

## Abstract

**Background:**

Maternal mortality remains a challenge in Ghana, where 263 women per 100 000 live births die during pregnancy or childbirth. Barriers to reaching the recommended antenatal care (ANC) include poor access to quality health care, cultural factors, and lack of support for pregnant women.

**Methods:**

We piloted two cross-randomized interventions: *durbars*, or local community meetings that incorporated education about ANC and supporting pregnant women, and an *enhanced ANC* model that added phone calls and a home visit to standard care. The study took place in 30 villages in the Upper East Region of Ghana between August 2021 and November 2022.

**Results:**

We tracked 277 women during pregnancy, with 120 women randomly assigned to the *enhanced ANC* intervention and 142 women living in villages randomized to the *durbar* intervention. Women who were randomized to the *enhanced ANC* intervention were 13.2 percentage points (pp) (95% confidence interval (CI) = 0.1, 24.3) more likely to have average or above average knowledge of pregnancy danger signs, 22.1 pp (95% CI = 9.1, 36.5) more likely to have a birth plan, 28.2 pp (95% CI = 13.0, 42.4) more likely to prepare the plan with their partners, and 16.4 pp (95% CI = 0.9, 29.3) more likely to pay for delivery with funds set aside in birth plan. They were also 35 pp (95% CI = 16.1, 48.1) more likely to make blood donor arrangements than control women who made birth plans. We found no impact of the *durbar* intervention on study outcomes.

**Conclusions:**

Evidence from this pilot suggests that interventions that increase interactions between health providers and pregnant women outside of the health facility may substantially improve women’s experience during pregnancy and maternal health outcomes. Providing structured ways for men to get engaged in ANC increased their involvement. Although we found no evidence the community meetings improved study outcomes, larger studies with repeated meetings and community-wide surveys are needed to make causal conclusions.

**Registration:**

American Economic Association RCT Registry: 10360; ISCRNT: ISRCTN95961119.

Maternal mortality remains a challenge in the Global South, with countries in sub-Saharan Africa being particularly affected. Almost all maternal deaths result from causes that could be averted with simple and low-cost interventions. The World Health Organization (WHO) recommendations for a healthy pregnancy include initiating antenatal care (ANC) in the first trimester, having at least eight contacts with health providers, delivering in a health facility, and initiating postnatal care within 24 hours of birth [1,2]. In Ghana, the maternal mortality rate of 263 women per 100 000 live births [3] far exceeds the Sustainable Development Goal 3.1 of <70 women per 100 000 [4]. Although access to health care has improved over time, many pregnant women still do not receive the recommended services [5–8]. Facilitating contact between providers and patients in rural and impoverished communities is essential to ensuring healthy pregnancies [5,6]. Interventions to improve women’s access to care need to incorporate socio-cultural factors such as lack of autonomy to make health decisions, especially when payment is required for services [9,10]. This calls for testing maternal interventions that are tailored to the context in which they are implemented and that do not exacerbate inequalities.

In Ghana, fees for maternal services were officially waived in 2004 [11], but many pregnant women continue to pay for care during pregnancy and delivery [6]. Indirect costs of attending ANC are also substantial: attending ANC is often an all-day ordeal that may require paying for transportation and food, as well as finding coverage for childcare and household chores. Since men typically control household finances, their minimal involvement in ANC and low opinion of the value of maternal health care contribute to underutilization of services [12]. Therefore, providing a structured way for them to become involved in pregnancy may improve women’s access to care. Birth preparedness – the process of planning for normal delivery and understanding how to identify and deal with complications – could offer an avenue for greater household engagement [13–15]. Incorporating intra-household dynamics and communicating directly with other household members may strengthen birth planning and the financial and moral support for women [14,16]. Male involvement during pregnancy is not the current social norm in Ghana [17]; changing perceptions about traditional gender roles may require the endorsement of messages around this topic from trusted authority figures [12,17]. Critically, involving other stakeholders in women’s health care needs to be done carefully to avoid potentially curtailing the limited autonomy women have in patriarchal societies [18].

Through this study, we aimed to strengthen the support for pregnant women and ease the burden of ANC seeking. We worked directly with communities to discuss the importance of ANC services and supporting women during pregnancy, and augmented standard ANC by adding home-based services that included monthly phone calls and a home visit to develop a birth plan together with other household members.

## METHODS

We implemented a cross-randomized trial to test the impact of two interventions. The first was the *durbar*, randomized at the community level, which provided an opportunity for health providers to meet with communities to discuss pregnancy and ANC. The second intervention was *enhanced ANC*, randomized at individual level, and delivered by adding monthly phone calls and a home visit to the standard ANC model. We chose the crossover design to enable us to pilot two interventions simultaneously with a limited sample, staff, and budget. We followed CONSORT guidelines for cross-randomized trials in reporting our findings [19].

### Study setting and timeline

**Figure 1:**
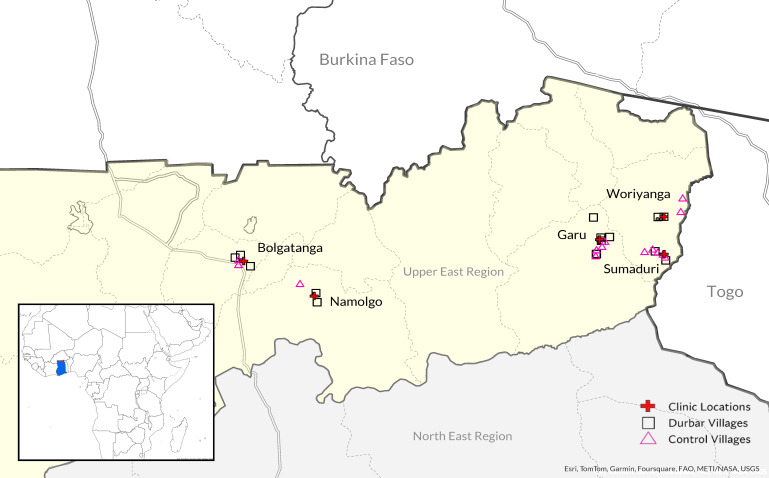
Map of Upper East Region of Ghana showing health facilities and villages included in the study. The map displays health facilities where the intervention was delivered (red cross), villages that were randomly assigned to *durbar* intervention (squares), and control villages where no *durbars* were held (triangles).

The study was set in the Upper East Region (UER) of Ghana, which is in the northeast corner of the country, near the border with Burkina Faso and Togo (**Figure 1**). This part of Ghana is highly impoverished: 76% of its population lives in extreme poverty (<USD 2/d), subsistence farming is the main economic activity, and fertility is high (4.1 in UER compared to 2.5 in Accra, the nation’s capital) [8,20,21]. We worked in 32 communities that are in the catchment area of the five Presbyterian Primary Healthcare Care (PPHC) centers that agreed to be a part of the pilot. The study sites included PPHC health centers in Garu, Sumaduri, Bolgatanga, Nomalgo, and Woriyanga. The study started with implementation of the *durbar* intervention (28 July to 8 August 2021), followed by six-month recruitment window (12 August 2021 to 13 January 2022), implementation of the *enhanced ANC* intervention and data collection (12 August 2021 to 31 August 2022), and qualitative interviews (November 2022).

### Study design and procedures

To implement the community-level intervention, our study leveraged *durbars*: traditional village meetings in northern Ghana where local authority figures discuss important matters with community members. *Durbars* are mobilized by the village chiefs and elders and the messages aired through these meetings are generally respected because they are sanctioned by the authority figures. We received permission to organize pregnancy-themed *durbars* in which the midwife from a local clinic met with men and women from the villages they serve to discuss the barriers women face to ANC attendance and provided education about pregnancy, ANC, and supporting women during pregnancy. Themes that were stressed by midwives to communities included the importance of starting ANC in the first trimester, the significance of various services included in ANC (such as hemoglobin test, ultrasound, iron supplements), and male partner involvement. The *durbar* intervention was delivered by midwives from the local clinic and attended by members of the research team. We randomized half of the communities in the study catchment area to receive an ANC-focused *durbar*. Randomization was allocated using a random number generator in Stata, with 50% chance of village selection for the intervention. Each randomly selected community received one ANC-themed *durbar* during the study period.

Following the *durbar* intervention, research staff were stationed at five PPHC clinics to recruit pregnant women for the study. Women were eligible for the study if they were 18 years or older, new registrants for ANC, and in the first or second trimester of pregnancy. Once recruited, research staff pulled a sealed envelope that assigned a random ID number and treatment assignment to the participant, with 40% chance of being allocated to the *enhanced ANC* intervention. The random ID numbers and treatment assignments were generated in Stata and sealed in the envelopes prior to study initiation. Control women received standard care that encouraged them to return to the health facility for in-person ANC services and deliver the baby at the health facility. Participants who were randomly allocated to the *enhanced ANC* intervention received standard care plus monthly phone calls and a home visit, which were delivered by midwives and community health officers (CHOs). Midwives made monthly phone calls, based on a schedule developed by the research staff, to check on the woman’s health and pregnancy, remind her of pregnancy danger signs, and encourage her to return for in-person checkups. CHOs added visits to pregnant women’s households to their routine community outreach trips. The home visits occurred in the seventh month of pregnancy to ensure the plans were already tangible but also left enough time to make the necessary arrangements. The CHOs scheduled the home visits ahead of time to maximize the chances that the pregnant woman’s husband and mother-in-law were available to take part in birth planning.

We collected two rounds of survey data (Table S1 in the **Online Supplementary Document**). Round 1 data, collected immediately after the first ANC visit, included baseline information about the woman’s individual and household characteristics, her previous pregnancy and childbirth experience, the first ANC visit experience, empowerment, and any information she may have recalled about pregnancy-related *durbars*. Round 2 data, collected within 4–8 weeks of giving birth, focused on the quality of ANC services, support, and decision-making during pregnancy, birth planning, childbirth experience, and empowerment. The empowerment scale included in the surveys was adopted from the Demographic Health Surveys [22]. The primary outcome variable to test the impact of the *durbar* intervention was whether a woman initiated ANC in the first trimester and the primary outcome to test the impact of the *enhanced ANC* intervention was whether she developed a birth plan.

The study concluded with qualitative interviews of households randomized to the interventions and the health providers involved in the study. Details about qualitative data methods are beyond the scope of this study and will be explored in future work.

The study procedures and analysis plan were pre-registered with the American Economics Association RCT Registry (#10360) and the ISRCTN (ISRCTN95961119).

### Statistical analysis

We estimated the impact of the interventions by fitting ordinary least squares models in which the outcomes were regressed on an indicator for the treatment assignment, catchment fixed effects, and a vector of individual and household characteristics: primiparity, age and age squared, education, partner's age and education (set to ‘missing’ category for unmarried women), religion, wealth index quintile. Separate models were fitted to estimate the impact of the *durbar* and *enhanced ANC* interventions. Standard errors were clustered at the community level and adjusted for small number of clusters using the wild bootstrap [23,24] (Appendix A: Research Methods in the **Online Supplementary Document**).

Sensitivity analyses included testing whether study results were robust to model specification by fitting logistic and modified Poisson regression models and estimating marginal effects with bootstrapped standard errors. We also made sure no catchment area was an outlier by testing whether omitting any catchment from the analysis changed study findings.

In power calculations, we set alpha at 0.05, power at 0.80, clusters at 32, and rho at 0.003. With these parameters and our plan to recruit 600 women, we had the power to detect differences of 11.7 percentage points (pp). We experienced slower than expected recruitment and our final sample includes 277 women, which diminished the study’s power.

We conducted the statistical analyses in Stata, version 16.1 (StataCorp LLC, College Station, Texas, USA), created the graphs in R, version 4.2.2 (R Core Team, Vienna, Austria), and generated the map in ArcGIS Pro, version 3.3 (Ersi, Redlands California, USA).

### Ethics statement

The Navrongo Health Research Centre Institutional Review Board (Ghana) and the University of California, Berkeley, Committee on Human Research (United States) approved the study procedures. Written informed consent was obtained from study participants.

## RESULTS

### Sample description

We collected data about 283 women from 30 communities. There were no new ANC registrants from two control villages during the recruitment period. Due to tablet malfunction, we lost survey responses from six women in round 2. The final analytic sample includes 277 women from 30 communities who have both rounds of data. 142 women lived in the communities randomized to the *durbar* intervention and 120 women were randomized to the *enhanced ANC* intervention.

The average participant was 27 years old, nearly all (99%) were married, 56% had primary education or less, and 29% were literate (**Table 1**). Most women in the sample (n = 179, 63%) had previously given birth. Descriptive characteristics of study participants were balanced at baseline in the *durbar* and control communities and the *enhanced ANC* and control women (Table S2, Panel A and B in the **Online Supplementary Document**). We detected some differences in how many women enrolled in ANC from the five study facilities.

**Table 1:** Descriptive characteristics of study sample (n = 277)*

Descriptive characteristics	
Age in years, x̄ (SD)	26.9 (6.6)
Married	275 (99.3)
Polygamous	46 (16.6)
Education categories	
*None*	107 (38.6)
*Primary*	47 (17.0)
*Junior secondary*	66 (23.8)
*Senior secondary or more*	57 (20.6)
Reads easily	81 (29.2)
Religion	
*Muslim*	104 (37.5)
*Christian*	170 (61.4)
*None*	3 (1.1)
Wealth quintiles	
*Poorest*	57 (20.6)
*Poor*	63 (22.7)
*Middle*	47 (17.0)
*Wealthy*	56 (20.2)
*Wealthiest*	54 (19.5)
Partner's age in years, x̄ (SD)	33.5 (8.1)
Partner's education	
*None*	105 (37.9)
*Primary*	27 (9.7)
*Junior secondary*	5 (1.8)
*Senior secondary or more*	137 (49.5)
Missing	3 (1.1)
Catchment	
*Bolga*	13 (4.7)
*Garu*	95 (34.3)
*Namolgo*	46 (16.6)
*Sumaduri*	71 (25.6)
*Woriyanga*	52 (18.8)
**Prior pregnancy information**	
Primiparity	101 (36.5)
Number of live births, x̄ (SD)†	2.8 (1.7)
Month ANC initiated last pregnancy, x̄ (SD)†	3.1 (1.4)
Number of ANC visits last pregnancy, x̄ (SD)†	6.2 (2.0)
All previous births in a facility†	145 (52.3)
Had postnatal care last pregnancy†	100 (36.1)
Had birth plan last pregnancy†	162 (92.6)

ANC – antenatal care, SD – standard deviation, x̄ – mean

*Presented as n (%) unless specified otherwise.

†Sample restricted to 176 women who had a previous pregnancy

Approximately half (53%) of the study participants initiated ANC in the first trimester, and the average woman in our sample had six ANC visits (**Table 2**; Table S4 in the **Online Supplementary Document**). Six women in the sample had miscarriages – four in the intervention and two in the control group. Nearly all participants (>98%) delivered their babies in a health facility and received postnatal care within 24 hours of the delivery. About a third of the women were the main decision makers to initiate ANC (34%) or were accompanied to ANC for their first ANC visit (29%). Furthermore, 32% of women in the sample received eight or more ANC visits as per WHO recommendations and 75% received all recommended ANC services during these visits. Most women (75%) developed a birth plan and 65% had their husbands help with the birth plan. The average woman paid about GH₵ 230, or roughly USD 30 (using 2022 exchange rate), in direct and indirect costs for the delivery (Table S4 in the **Online Supplementary Document**). About half (51%) of women paid for delivery with savings from the birth plan and 42% needed to borrow money or sell things to pay for the delivery.

**Table 2:** Summary statistics of outcome variables (n = 277), presented as n (%)*

Outcome measures	
*Durbar *intervention	
Initiated ANC in first trimester	147 (53.1)†
Decided on her own to come to first ANC	95 (34.3)
Accompanied by husband to first ANC	81 (29.2)
***Enhanced ANC* intervention**	
≥8 ANC visits	89 (32.1)
Received recommended ANC services during pregnancy	208 (75.1)
Average or above knowledge of danger signs	180 (65.0)
Woman chose ANC frequency	94 (33.9)
Husband came to ANC	124 (44.8)
Developed birth plan	208 (75.1)†
*Used birth plan*	199 (71.8)
*Husband helped with birth plan*	180 (65.0)
*Paid for delivery using savings from birth plan*	140 (51.1)
*Borrowed money or sold possessions to pay for delivery*	114 (42.2)

ANC – antenatal care

*Table S5 in the **Online Supplementary Document** also includes summary statistics of continuous measures.

†Main outcome measures.

### Interventions

We conducted 16 *durbars* in the communities randomly selected from the catchment areas. The *durbar* intervention participants identified poverty, lack of knowledge about when to initiate ANC, long distance to health facility, disrespect from providers, and inadequate spousal support as the main barriers to ANC. The issue of spousal support often generated heated debates between attendees, with men generally stating they already provided support, and women voicing that the support was inadequate (e.g. not enough money to pay for tests) and mistimed (e.g. supporting them only during labor).

**Figure 2:**
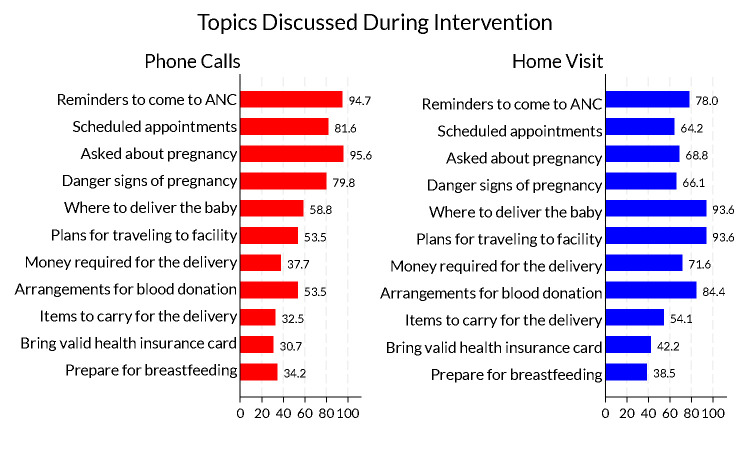
Topics discussed during *enhanced ANC* intervention based on a sample of 120 women randomly assigned to the *enhanced ANC* intervention. Each bar represents the proportion of respondents in the *enhanced ANC* intervention who said they discussed the topics during phone calls and home visits.

Phone conversations were largely general check-ins about the pregnancy (95.6%), reminders to come to ANC for a physical visit (94.7%), scheduling appointments (81.6%), and reminders about pregnancy danger signs of (79.8%). Home visits focused on the upcoming delivery: where to deliver the baby (93.6%), plans for traveling to the health facility during labor (93.6%) and arranging for blood donors (84.4%) (**Figure 2**).

### Impact of the *durbar* and *enhanced ANC* interventions

**Figure 3:**
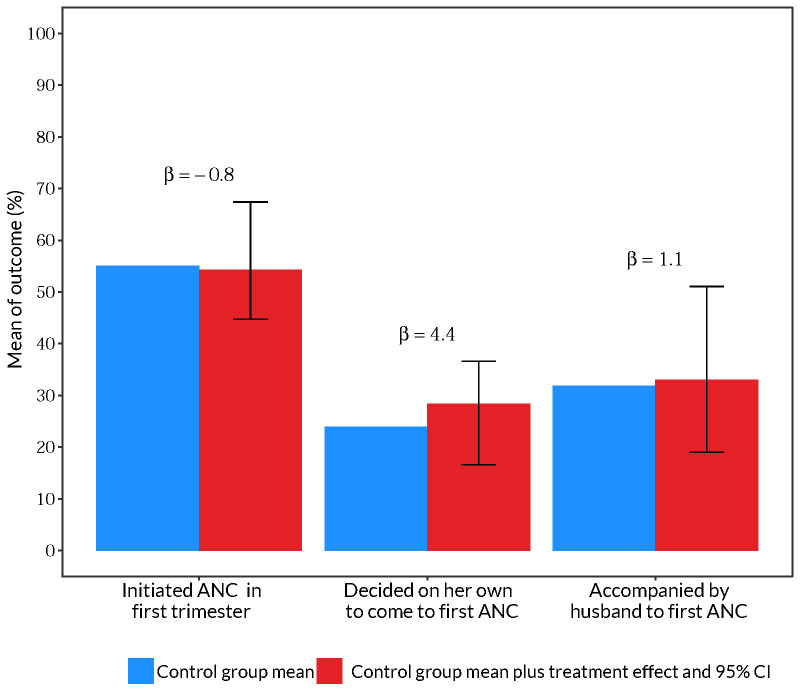
No evidence the *durbar* intervention affected study outcomes related to first ANC visit based on 277 women participating in the study. Blue bars represent the control group mean. Red bars represent the control group mean plus treatment effect of the *durbar* intervention. The treatment effect, which can be interpreted as pp difference from the control mean, is also reported as β above the bars. The error bars are 95% CIs around the treatment effects. The outcomes are labeled below bars along the x-axis. Each outcome was estimated using a separate regression model that controlled for primiparity, age and age squared, education, partner's age and education, religion, wealth index quintile, and catchment fixed effects. Standard errors were clustered at the village level and bootstrapped by the wild method. Regression results are also presented in Table S6 in the **Online Supplementary Document**

We found no impact of the *durbar* intervention on study outcomes, including when participants started ANC (**Figure 3**; Table S6 in the **Online Supplementary Document**). Only 20.1% of women from *durbar* intervention villages attended the meetings (Table S2 in the **Online Supplementary Document**). Women from *durbar* villages were 4.4 pp more likely to be the primary decision maker on when to initiate ANC, difference not statistically significant (95% confidence interval (CI) = −7.1, 12.9).

**Figure 4:**
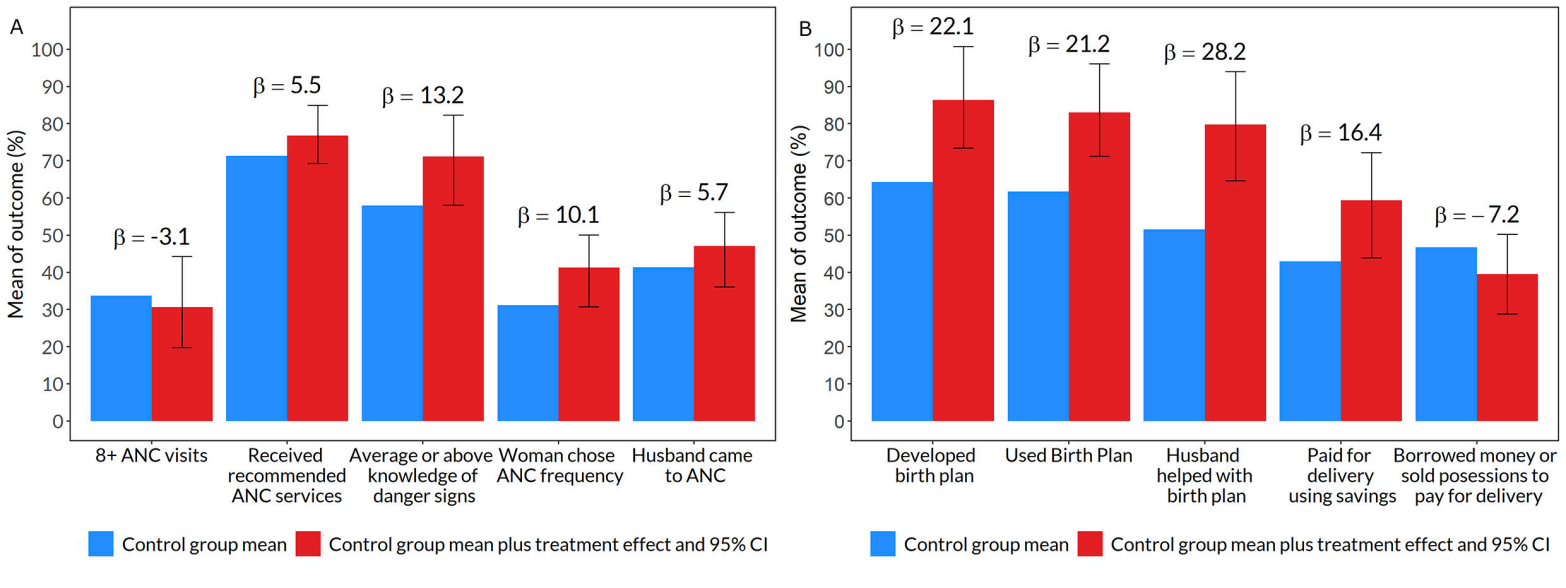
Impact of *enhanced ANC* intervention on study outcomes based on 277 women participating in the study. Blue bars represent the control group mean. Red bars represent the control group mean plus treatment effect of the *durbar* intervention. The treatment effect, which can be interpreted as pp difference from the control mean, is also reported as β above the bars. The error bars are 95% CIs around the treatment effects. The outcomes are labeled below bars along the x-axis. Each outcome was estimated using a separate regression model that controlled for primiparity, age and age squared, education, partner's age and education, religion, wealth index quintile, and catchment fixed effects. Standard errors were clustered at the village level and bootstrapped by the wild method. Regression results are also presented in Table S7 in the **Online Supplementary Document**. **Panel A.** *Enhanced ANC* improved knowledge of danger signs and participation in medical decisions. **Panel B.** *Enhanced ANC* improved birth preparedness.

The *enhanced ANC* intervention did not affect the number of ANC visits or the likelihood of receiving the recommended ANC services (**Figure 4**, Panel A; Table S6 in the **Online Supplementary Document**). Women assigned to the *enhanced ANC* intervention were 13.2 pp (95% CI = 0.1, 24.3) more likely to have average or above average knowledge of pregnancy danger signs and 10.1 pp (95% CI = −0.5, 18.9) more likely to be the primary decision-maker about ANC frequency. Intervention women were also 5.7 pp (95% CI = −5.3, 14.7) more likely to come to ANC with their husbands, but this difference was not statistically significant.

The *enhanced ANC* intervention significantly improved birth preparedness (**Figure 4**, Panel B; Table S7 in the **Online Supplementary Document**). Women randomized to *enhanced ANC* were 22.1 pp (95% CI = 9.1, 36.5) more likely to have a birth plan, 28.2 pp (95% CI = 13.0, 42.4) more likely to develop the plan with their husbands, 16.4 pp (95% CI = 0.9, 29.3) more likely to pay for delivery from savings, and 7.2 pp (95% CI = −18.0, 3.5) less likely to have to borrow money or sell possessions to pay for the delivery.

We found no evidence that the *enhanced ANC* intervention affected women’s empowerment (Table S14 in the **Online Supplementary Document**).

### Components of birth plans

**Figure 5:**
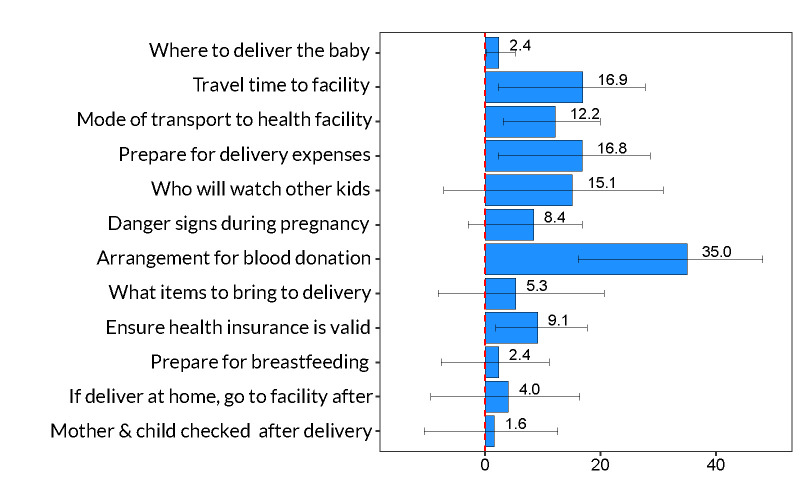
Components of birth plans for women randomized to *enhanced ANC* intervention compared to control women. Based on 208 women participating in the study who developed birth plans. Each bar represents the treatment effect of the *enhanced ANC* intervention, which can be interpreted as pp change from the control mean women. 95% CIs calculated around the treatment effect. Each birth plan component was estimated in a separate regression model that was regressed on a binary indicator for *enhanced ANC* intervention, conditional on having a birth plan, and controlled for primiparity, age and age squared, education, partner's age and education, religion, wealth index quintile, and catchment fixed effects. Robust standard errors were bootstrapped by the wild method.

Conditional on having a birth plan, women randomized to the *enhanced ANC* intervention were significantly more likely to plan and execute the components of their birth plans that involved logistics of travel to the health facility, payment for delivery, having valid health insurance prior to delivery, and arrangements for blood donations (**Figure 5**). Women in the *enhanced ANC* intervention were 35.0 pp (95% CI = 16.1, 48.1) more likely to make arrangements for blood donors, which translates to a 125% increase in absolute terms, from 28.0% of women in the control group to 63.0% of women in the *enhanced ANC* intervention.

### Robustness checks

The study findings were robust to fitting modified Poisson and logistic regression models instead of ordinary least squares (Tables S8–11 in the **Online Supplementary Document**). We made sure no study site was an outlier by leaving out each catchment area from the analysis (Tables S12 and S13 in the **Online Supplementary Document**). There is suggestive evidence that the *enhanced ANC* intervention may have been most effective in the Garu catchment area.

## DISCUSSION

Our study piloted interventions that aimed to improve women’s access to health providers during pregnancy through services that incorporated their husbands and mothers-in-law. We worked in the northern, rural part of Ghana where access to high quality maternal services is low, poverty is high, and many women lack the autonomy to make independent decisions about their health care. We incorporated local beliefs and traditions in our design to ensure acceptability and fit of the interventions. The *durbar* intervention offered a forum for community members to discuss the barriers to ANC and support for pregnant women. The *enhanced ANC* model provided repeated interactions with health providers and directly incorporated other household members in pregnancy.

We found a strong signal that the *enhanced ANC* intervention improved women’s knowledge and birth preparedness. The phone calls provided an opportunity for providers to repeat the education multiple times, and the home visit directly involved other household members in birth planning. Similar to other contexts [25,26], the men who participated in the *durbar* intervention expressed a desire to support women but did not necessarily know *how* or *when* to get involved. Creating structured opportunities for men to become involved appears to be important. The process of birth planning includes many financial decisions such as setting aside money for delivery-related expenses and making blood donor arrangements, as it requires paying for laboratory expenses to identify a donor who matches the pregnant woman’s blood group [13–15]. We found that engaging men in the process increased the likelihood that birth plans were implemented and concentrated the plans on critical components. Intervention women were more than twice as likely to make blood donor arrangements compared to control women. Haemorrhage is the top cause of maternal mortality globally, accounting for 27.1% of maternal deaths [21]; being prepared to address this complication is crucial to reducing maternal mortality.

In patriarchal societies such as northern Ghana, men control household decisions, especially those involving money. Despite maternal health services technically being free in Ghana, women in our sample reported spending about USD 30 on in-facility delivery (including laboratories, medications, supplies, transport, food, etc.), a cost that can be catastrophic for households living in extreme poverty [20,21]. Women who received *enhanced ANC* were significantly more likely to pay for delivery with money set aside in birth plans and less likely to have to borrow money or sell things to pay for the delivery. An additional benefit of dyadic birth planning is the potential to improve couples’ communication and marital satisfaction, as has been found in a Zambian study that addressed couples’ discordance in fertility preferences [27]. The experiment varied whether information about risks of maternal mortality was delivered to men or women and found that educating husbands led to higher birth spacing and reduced fertility, closer in line with the woman’s fertility preferences. Sharing information directly with men was more effective than relying on wives to communicate the information with their spouses. Similarly, we found that incorporating male partners directly has led to more effective birth planning, including critical components of birth plans such as arranging a blood donor and setting aside funds for the upcoming birth. Finding additional ways to involve men, such as educating them about pregnancy danger signs, importance of supplements, or proper diet during pregnancy, may be highly effective in changing household behaviors.

Although we found no evidence that the *durbar* intervention affected outcomes related to ANC initiation, the results should be interpreted with caution due to methodological limitations. We implemented the *durbar* intervention once per community and measured the impact only among pregnant women. Consistent with other studies, we found that incorporating men in pregnancy is often at odds with deeply entrenched social norms [18]. We aimed to address this issue by incorporating local authority figures who wield significant influence in the meetings, but the intervention may not have been intensive enough to make an impact. The preliminary findings from our qualitative interviews suggest strong support for pregnancy-related *durbars* among community members who were eager to share their experiences with the health care system. Health workers also reported using the feedback from the community to improve service delivery. Providing an avenue for communities to meet regularly to discuss maternal health with providers may build trust and offer important benefits to all parties [28,29].

The study was subject to limitations. The recruitment of participants was slower than anticipated; we initially aimed to recruit 600 participants, but were only able to recruit 283 women, and six Round 2 responses were lost due to tablet malfunction, which brought our final analytic sample to 277 women who had data from both survey rounds. This small sample diminished our power to detect statistical differences and we were therefore unable to test whether receiving both interventions affected our study outcomes. We also did not have sufficient sample size to estimate whether providing only the phone calls or only the home visit may have improved the study outcomes on their own.

We measured the impact of the intervention using self-reported data, which are subject to social desirability and recall bias. We aimed to address this issue by asking the questions soon after first ANC visit and soon after giving birth to improve recall and conducting the interview in a local language by trained enumerators who were native to the study setting to address social desirability bias.

We also detected a baseline imbalance in facility catchment areas which we attribute to the small number of women in our sample. To address this, we included a catchment covariate in all regressions and confirmed that omitting a catchment area from the analysis did not meaningfully change study findings.

We conducted only one *durbar* per community, and some meetings had limited attendance because the community members were busy with farm work. The meetings included both men and women and this could have dissuaded some participants from discussing pregnancy and gender roles. An important part of the intervention was addressing the social norm of limited male involvement in pregnancy, and we believe that it was essential for the intervention to be delivered in an open forum with both men and women present. Further understanding of the role of male participation in pregnancy is important and larger studies should include direct measures of men’s attitudes and beliefs on this topic.

## CONCLUSIONS

Our findings show that connecting women to health providers throughout their pregnancies and involving their husbands in care improves pregnancy experience. Women were clear during the *durbars*: they want more help during pregnancy, and they think the help should come from their partners. Offering structured ways for men to assist women during pregnancy, such as help with birth preparedness, could significantly improve women’s well-being and possibly also increase cohesion among couples.

## Additional material


Online Supplementary Document
