# Supplementary material for: It takes a village: A pilot cross-randomized trial to enhance pregnancy care and support in northern Ghana
Source: J Glob Health. 2024 Oct 25;14:04217. doi: 10.7189/jogh.14.04217 (PMC11506688; doi:10.7189/jogh.14.04217)
Supplement: Online Supplementary Document [file jogh-14-04217-s001.pdf]

It Takes a Village: A Pilot Cross-Randomized Trial to Enhance Pregnancy Care and Support in Northern Ghana

Jakubowski, A, Aborigo, RA, Kuwolamo, I, Meredith, JD, and Aubosi, AA.

Supplementary Appendix

Table of Contents

Appendix A: Research Methods 2

Study Design Overview 2

Interventions 2

Randomization 3

Data Collection Instruments 3

Round 1 Survey 3

Round 2 Survey 3

Other data 3

Empirical Specifications 4

Analysis variables 5

Outcome variables 5

Explanatory variables 5

Sample size 7

Appendix B: The CONSORT Flowchart 8

Appendix C: Balance at baseline 9

Appendix D: Adherence to study protocols 10

Appendix E: Pregnancy, delivery, and postnatal care experience 11

Appendix F: Detailed summary of outcome variables 12

Appendix G: Regression results in table format 13

Appendix H: Robustness Checks – Nonlinear Models 14

Appendix I: Robustness Checks – Leave-one-out Analysis 16

Appendix J: ANCOVA for Empowerment Outcomes 18

Appendix A: Research Methods

Study Design Overview

Our study is designed to test whether education at community meetings (called durbars in this setting) to communicate the importance of timely and frequent prenatal care, and individual education through phone cars and home visits that incorporate male partners increase the use of maternal health services and improve health outcomes in northern Ghana. The general outline of our interventions and data collection procedures is as follows:

1. Random assignment of which communities receive antenatal care (ANC) messages at durbars.
2. Intervention 1: Education at durbars.
3. Recruit and enroll pregnant women in study at 1st antenatal visit.
4. Randomize individuals to receive phone calls and a home visit.
5. Conduct baseline survey (in person).
6. Intervention 2: Phone calls (monthly) and home visit (one time).
7. Conduct endline survey (in person unless women express preference for phone interview).

Interventions

The intervention involved two cross-randomized interventions: 1) community education at **Durbars**, 2) **Enhanced ANC** model during which monthly phone calls and one home visit were made to reinforce key messages, monitor pregnancy, and prepare birth plans together with husbands and mothers-in-law.

Durbars are regular village meetings where communities discuss important village matters. Since durbars take place on a usual basis, nurses coordinated with the local chiefs and elders to pre-arrange durbars to deliver the interventions. The chiefs and elders in turn employed their mechanisms of organizing durbars to invite the community members to participate. The key quality of durbars that we leveraged in our intervention is that these are ongoing meetings that happen on a regular basis and that involve village chiefs and other local authority figures, whose endorsement of the messages encouraging early ANC and male involvement in their wives' healthcare may cause them to be taken more seriously. We sought the endorsement of our messages from the chiefs and elders prior to the durbars, and the intervention was randomized across villages where the leaders were willing to hold a durbar focused on ANC.

Attendance at the durbars was completely voluntary and was not monitored by research staff - community members came to these meetings when they wished, and they were free to leave whenever they deemed appropriate. We tested the impact of providing education about the importance of starting ANC in the first trimester by comparing whether the month of pregnancy when women first presented for ANC changed in the villages that held durbars vs. those that did not.

After durbars were held, the study staff began enrolling women in the five study facilities. Women who agreed to participate were provided informed consent, were randomized to receive either routine care or the **Enhanced ANC** intervention which included basic ANC, monthly phone calls, and one home visit

Monthly calls: the calls were made by health center staff to follow up on the progress of the pregnancy, encourage women to come to the facility for in-person ANC check, and develop a birth preparedness plan. The innovation of this treatment is that instead of receiving pre-recorded messages (i.e. the CHPS program), women were connected with a midwife who can go over any possible complications and encouraged them to go in for a check-up. This activity was conducted by the nurses/midwives. Research staff were not involved in this activity.

Home visit: in the Enhanced ANC treatment group received one household visit from a community health officer (CHO) to develop a concrete birth preparedness plan with the pregnant woman and her household members. The health provider arranged a time to visit the household when either the woman's husband or mother-in-law (ideally both) were at home, ensuring that the families developed a plan together. The health provider prompted the households to make specific choices for the plan, such as: identifying danger signs during pregnancy and labor, choosing the health facility where the woman can deliver, determining how long it takes to travel there, making specific plan on how they will travel there, establishing how much money was required for the birthing process, and planning how to set aside money for this expense. This activity was conducted by the nurses/midwives. Research staff were not involved in this activity.

Randomization

Randomization to the durbar treatment was at the village level; randomization to the phone call and home visit treatments was at the woman-level. The cross-randomization was to enable us to tease out the specific aspects of these intervention on outcomes of interest. Women who were randomized to the pure control arm received standard care: during each contact with health providers they were encouraged to return for at least eight ANC contacts, deliver in a health facility, and have a postnatal check.

Data Collection Instruments

Data collection relied on in-person surveys conducted with the study participants at enrollment (co-occurring with 1st ANC visit) and after delivery of the baby (max 8 weeks after delivery). Participants were asked to reiterate their consent prior to conducting the interview. Both rounds were conducted in person, with the former at a healthcare facility and the latter in the participant's home.

Round 1 Survey

The Round 1 questionnaire gathered descriptive information about the participant and her household as well as detailed questions about her current and previous pregnancies. The survey was run from August 12, 2021 to January 13, 2022. List of the topics covered is as follows: Demographics, Household characteristics, Pregnancy and birth history, Current pregnancy, Beliefs about ANC start time, Durbar questions, Male partner involvement, Community involvement, Women empowerment**.**

Round 2 Survey

The Round 2 questionnaire gathered detailed information about the current pregnancy, the care that women received, and pregnancy outcomes. The survey was run from February 5, 2022 to August 31, 2022. List of the topics covered is as follows: Demographics, Quality of ANC, Birth plan, Birth experience, Male partner involvement, Women empowerment.

Other data

Program implementation data: We tracked whether women randomized to the intervention arm were receiving the phone calls and home visits as planned.
Qualitative data: We interviewed a small number of women and all providers about their experiences with the study.

Empirical Specifications

We conducted the analysis in two stages. The first stage entailed testing whether the village level treatment assignment to durbars affects outcomes. The second entails testing whether the individual level assignment to phone calls and home visits affects outcomes.

Using Round 1 data we will estimate model specified in Equation 1:

(1) $Y_{iv} = \alpha + \beta\ Durbar_v + X'_{iv}\delta + HC_v\ \mu + \epsilon_{iv}$

where Y_{iv} is the outcome of individual i who lived in village v. $Durbar_v$ is an indicator variable for whether individuals in village v received durbar intervention. X_{iv} is a vector of individual-level covariates (e.g. age, level of education, primiparity, household wealth, timing of previous ANC) that we will control for in case we have any imbalance issues since we did not collect baseline data. Standard errors are clustered at the village level and bootstrapped by the Wild method. HC_v is an indicator for the health clinic that is near the study village in case that is an important determinant of whether the durbar (led by midwife from that clinic) had an impact on study outcomes.

Next, to evaluate the impact of the Enhanced ANC intervention (phone calls and home visit) which was randomized at the individual level, we will fit the model specified in Equation 2:

(2) $Y_{i,t=1} = \alpha + \beta\ EnhancedANC_i + X'_{i,\, t=0}\delta + HC_v\mu + \epsilon_i$

where Y is the outcome of individual *i*. $EnhancedANC_i$ is an indicator variable for whether individuals were randomized to the Enhanced ANC intervention (phone call and home visit) or if they were in the control group. X_{iv} is a vector of individual-level covariates collected at enrollment (e.g. age, level of education, primiparity, household wealth). HC_v is an indicator for the health clinic that is near the study village in case the quality of the intervention differed by facility (phone calls and home visits were delivered by midwives and CHOs from the respective health centers).

The *Durbar* and *Ehanced ANC* interventions were cross-randomized, and ideally we would like to evaluate whether receiving both interventions had a larger impact of study outcomes. However, we were unable to run this model because of the very limited sample size of the pilot study.

Analysis variables

Outcome variables

All outcome variables were pre-specified in the analysis plan unless otherwise noted. The outcomes for the *Durbar* intervention were related to ANC initiation: the month of pregnancy at first ANC, whether ANC was initiated in the first trimester, the number of days between knowing about pregnancy and initiating ANC, whether women initiated ANC according to their own preferences, and whether the first ANC visit was for a routine checkup.

The outcome variables related to the *Enhanced ANC* intervention can be grouped into three themes. 1) **Quality of ANC services:** number of in-facility visits, number of ANC services provided, number of danger signs known, and above average knowledge of danger signs; 2) **Support and decision making during pregnancy:** woman was the primary decision maker about ANC, someone accompanied her to ANC, her husband/partner accompanied her to ANC, woman felt supported by her community and by her husband/partner, husband/partner was helpful during pregnancy, and mother-in-law was involved in woman's pregnancy care; 3) **Birth planning and delivery:** developed a birth plan, used the birth plan, family members helped with birth planning, husband accompanied her to the delivery, woman paid for delivery using savings from birth plan, woman borrowed money or sold things to pay for the delivery. Finally, we assessed women's empowerment using a scale adopted from the Demographic Health

Explanatory variables

The analysis included two explanatory variables related to treatment assignment. Durbar variable was set to 1 if a woman lived in a community that was randomized to receive *Durbar* intervention. Enhanced ANC variable was set to 1 if a woman was randomized to the *Enhanced ANC* intervention. The models also included a vector of individual characteristics of participants: primiparity, age and age squared, education, partner's age and education (set to missing for unmarried women), religion, wealth index quintile, and catchment fixed effect.

For simplicity and ease of interpretation, the outcome variables presented in the main manuscript are binary measures based on the variables we describe above. For example, "month of pregnancy at first ANC" is less meaningful than the binary indicator "woman initiated ANC in first trimester". We present the models using continuous measures in the Supplement and find results that are consistent with the binary outcome models.

Supplementary Appendix

Table S1
Study outcomes were pre-registered in the pre-analysis plan. We created binary variables based on the pre-registered outcomes to ease analysis and interpretation of the results. We also added measures about financial planning during birth plans and whether husband accompanied the respondent to her first ANC visit.

Definition	Survey	Name	Type	Definition	Analysis	Registered
Outcome	Round 1	Month of pregnancy at 1st ANC	Numeric	Month of pregnancy at 1st ANC.	Impact of *Durbar*	Yes
Outcome	Round 1	ANC initiated in 1st trimester	Binary	ANC was initiated in the 1st trimester.	Impact of *Durbar*	Yes
Outcome	Round 1	Decided on her own to come to ANC	Binary	Participant made the decision to come to 1st ANC herself (ref: others decided for her)	Impact of *Durbar*	Yes
Outcome	Round 1	Accompanied by husband to first ANC	Binary	Participant's husband came with 1st ANC visit	Impact of *Durbar*	No
Independent	Round 1	Durbar	Binary	Participant is from a village randomized to the *Durbar* intervention.	Impact of *Durbar*	Yes
Outcome	Round 2	# ANC visits	Numeric	Number ANC visits during pregnancy.	Impact of *Enhanced ANC*	Yes
Outcome	Round 2	Had 8 or more ANC visits	Binary	Based on number ANC visits during pregnancy.	Impact of *Enhanced ANC*	No
Outcome	Round 2	# ANC services	Numeric	Number of ANC services received during the pregnancy. Max of 10.	Impact of *Enhanced ANC*	Yes
Outcome	Round 2	Received the recommended ANC services	Binary	Based on number of ANC services received during the pregnancy.	Impact of *Enhanced ANC*	No
Outcome	Round 2	# Danger Signs Known	Numeric	Number of pregnancy danger signs the participant could list when asked. Max of 9.	Impact of *Enhanced ANC*	Yes
Outcome	Round 2	Average or above average knowledge of danger signs	Binary	Participant can identify at least 4 danger signs.	Impact of *Enhanced ANC*	No
Outcome	Round 2	Woman primary decision maker on ANC frequency	Binary	Participant was the primary decision maker on how often to go to ANC.	Impact of *Enhanced ANC*	No
Outcome	Round 2	Husband accompanied to ANC	Binary	Participant's husband ever came to ANC.	Impact of *Enhanced ANC*	Yes
Outcome	Round 2	Felt supported by husband	Binary	Participant felt supported or very supported during pregnancy, using a 5 point Likert scale.	Impact of *Enhanced ANC*	Yes
Outcome	Round 2	Had birth plan	Binary	Participant developed a plan for her delivery.	Impact of *Enhanced ANC*	Yes
Outcome	Round 2	Used birth plan	Binary	Participant used her birth plan for delivery.	Impact of *Enhanced ANC*	Yes
Outcome	Round 2	Husband helped with birth plan	Binary	Participant's husband involved in developing birth plan.	Impact of *Enhanced ANC*	Yes
Outcome	Round 2	Paid for delivery using savings from birth plan	Binary	Participant used money set aside in birth plan to pay for delivery.	Impact of *Enhanced ANC*	No
Outcome	Round 2	Borrowed money or sold things to pay for delivery	Binary	Participant borrowed money or sold possessions to pay for delivery.	Impact of *Enhanced ANC*	No
Outcome	Round 2	Husband came to delivery	Binary	Participant's husband attended delivery.	Impact of *Enhanced ANC*	Yes
Independent	Round 2	Enhanced ANC	Binary	Participant was randomized to *Enhanced ANC* treatment	Impact of *Enhanced ANC*	Yes

Sample size

The study was designed to recruit women for a 6 month period with the aim of recruiting 600 pregnant women during the enrolment period. In our power calculations, we set alpha at 0.05, power at 0.80, clusters at 30, and rho at 0.003. With these parameters, we had the power to detect differences of 11.67 percentage points. Power calculations were made in Stata 18 using the “power twoproportions” command.

Appendix B: The CONSORT Flowchart

We collected data about 283 women from 30 communities. There were no new registrants for ANC during the recruitment period from two control villages. Due to tablet malfunction, we lost survey responses from six women in Round 2. The final analytic sample includes 277 women from 30 communities who have both rounds of data. 142 women lived in the communities randomized to the Durbar intervention and 120 women were randomized to the Enhanced ANC intervention.

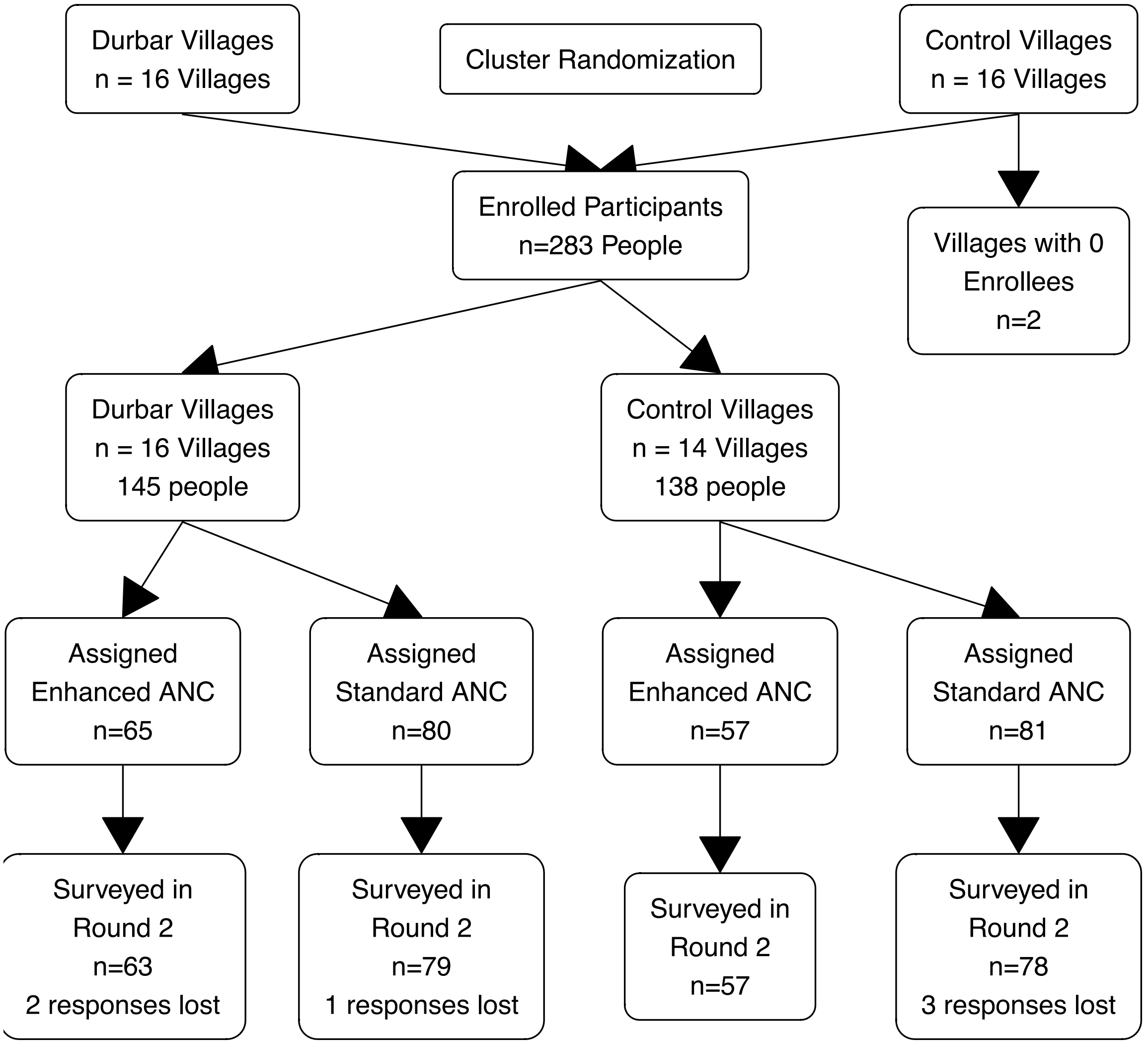

Appendix C: Balance at baseline

Table S2: Descriptive characteristics of sample balanced at baseline

	Panel A: *Durbars* Intervention (village-level randomization)		Panel B: *Enhanced ANC* Intervention (individual-level randomization)	
**Treatment Assignment**	Control villages	*Durbars* villages	Control women	*Enhanced ANC* women
	N=135 (49%)	N=142 (51%)	N=157 (57%)	N=120 (43%)
Descriptive characteristics				
Age	26.4 (6.4)	27.4 (6.8)	26.7 (6.7)	27.2 (6.6)
Married	135 (100%)	140 (98.6%)	156 (99.4%)	119 (99.2%)
Polygamous	26 (19.3%)	20 (14.1%)	29 (18.5%)	17 (14.2%)
Education categories				
None	49 (36.3%)	58 (40.8%)	68 (43.3%)	39 (32.5%)
Primary	24 (17.8%)	23 (16.2%)	27 (17.2%)	20 (16.7%)
Junior Secondary	32 (23.7%)	34 (23.9%)	31 (19.7%)	35 (29.2%)
Senior Secondary or More	30 (22.2%)	27 (19.0%)	31 (19.7%)	26 (21.7%)
Reads easily	48 (35.6%)	33 (23.2%)	40 (25.5%)	41 (34.2%)
Religion				
Muslim	51 (37.8%)	53 (37.3%)	58 (36.9%)	46 (38.3%)
Christian	82 (60.7%)	88 (62.0%)	96 (61.1%)	74 (61.7%)
None	2 (1.5%)	1 (0.7%)	3 (1.9%)	0 (0.0%)
Wealth Quintiles				
Poorest	33 (24.4%)	24 (16.9%)	36 (22.9%)	21 (17.5%)
Poor	26 (19.3%)	37 (26.1%)	36 (22.9%)	27 (22.5%)
Middle	22 (16.3%)	25 (17.6%)	28 (17.8%)	19 (15.8%)
Wealthy	26 (19.3%)	30 (21.1%)	28 (17.8%)	28 (23.3%)
Wealthiest	28 (20.7%)	26 (18.3%)	29 (18.5%)	25 (20.8%)
Partner's Age	33.4 (7.6)	33.6 (8.7)	33.5 (8.1)	33.4 (8.2)
Partner's Education				
None	47 (34.8%)	58 (40.8%)	64 (40.8%)	41 (34.2%)
Primary	13 (9.6%)	14 (9.9%)	16 (10.2%)	11 (9.2%)
Junior Secondary	3 (2.2%)	2 (1.4%)	2 (1.3%)	3 (2.5%)
Senior Secondary or More	71 (52.6%)	66 (46.5%)	73 (46.5%)	64 (53.3%)
Missing	1 (0.7%)	2 (1.4%)	2 (1.3%)	1 (0.8%)
Catchment				
Bolgatanga	2 (1.5%)	11 (7.7%)	6 (3.8%)	7 (5.8%)
Garu	47 (34.8%)	48 (33.8%)	50 (31.8%)	45 (37.5%)
Namolgo	13 (9.6%)	33 (23.2%)	30 (19.1%)	16 (13.3%)
Sumaduri	46 (34.1%)	25 (17.6%)	43 (27.4%)	28 (23.3%)
Woriyanga	27 (20.0%)	25 (17.6%)	28 (17.8%)	24 (20.0%)
Previous pregnancy experience				
Primiparity	50 (37.0%)	51 (35.9%)	58 (36.9%)	43 (35.8%)
Number of live births	2.7 (1.7)	2.9 (1.7)	2.9 (1.7)	2.7 (1.7)
Month ANC initiated last pregnancy	3.1 (1.4)	3.0 (1.5)	3.0 (1.3)	3.1 (1.6)
Number of ANC visits last pregnancy	6.2 (2.0)	6.3 (2.0)	6.2 (2.0)	6.4 (2.0)
All previous births in facility	70 (51.9%)	75 (52.8%)	80 (51.0%)	65 (54.2%)
Had postnatal care last pregnancy	53 (39.3%)	47 (33.1%)	57 (36.3%)	43 (35.8%)
Had birth plan last pregnancy	82 (96.5%)	80 (88.9%)	90 (91.8%)	72 (93.5%)

Notes: Based on information from Round 1 survey. Based on data from 277 women who appear in both Round 1 and Round 2 surveys. Each cell contains the mean and standard deviation in parentheses for numeric variables and count and frequency in parentheses for binary variables.

Appendix D: Adherence to study protocols

We find limited participation in durbars among study participants and strong adherence to study protocols in the *Enhanced ANC* intervention. Only 55.4% of women from durbar villages recall having a durbar related to ANC in their villages and 20.1% reported attending the durbar (Appendix Table 3). Nearly all women randomized to enhanced ANC intervention reported receiving calls (95.7%) and home visits (91.5%). Three women (2.2%) from control villages recalled attending a durbar. One control woman (<1%) recalled receiving calls and two women in the Enhanced ANC control group (1.3%) recalled a home visit.

Table S3. Adherence to Study Protocols

**Panel A: Durbar intervention**	Control	Intervention
	N=135	N=142
Recalls the Durbar	3 (2.2%)	80 (56.3%)
Attended the Durbar	3 (2.2%)	31 (21.8%)

**Panel B: Enhanced ANC intervention**	Control	Intervention
	N=157	N=120
Call intervention		
Received Calls from Midwife	1 (0.6%)	**114 (95.0%)**
Number of Calls Received	–	5.5 (1.5)
Frequency of Calls		
Too many	–	4 (3.5%)
Just right amount	–	55 (48.2%)
Too few	–	55 (48.2%)
Wants to receive calls if pregnant again	–	114 (100.0%)
Home visit intervention		
Home visit by Community Health Officer	2 (1.3%)	**109 (90.8%)**
Month of pregnancy when received home visit	–	6.6 (0.6)
Home visit scheduled ahead of time	–	107 (98.2%)
Someone else at home during visit	–	102 (93.6%)
Who was present at home visit		
Husband	–	94 (86.2%)
Mother-in-law	–	76 (69.7%)
Other relatives of friends	–	63 (57.8%)
Who was involved in discussions during home visit		
Husband	–	92 (84.4%)
Mother-in-law	–	76 (69.7%)
Other relatives of friends	–	41 (37.6%)
Believes others would benefit from home visit	–	91 (75.8%)
Wants home visit if pregnant again	–	109 (100.0%)
Wants home visit with other family members if pregnant again	–	105 (96.3%)

[a] Restricted to participants present in both rounds of the survey. Percentages are out of the entire treatment group or control group. Abbreviations: ANC = Antenatal Care

Appendix E: Pregnancy, delivery, and postnatal care experience

The women in our sample had an average of six ANC visits (Appendix Table 3). Six women in the sample had miscarriages: 4 women in the intervention arm and 2 in the control arm. Nearly all participants (>98%) delivered their babies in a health facility and received postnatal care within 24 hours of the delivery. Women spent on average 40 minutes traveling to the health facility for delivery with most of them (83%) doing so on a motorbike. The average woman paid nearly US$30 in total costs for the delivery.

Table S4. Pregnancy, delivery, and postnatal care experience

	Control	Intervention
	N=157	N=120
Antenatal care experience		
Number of ANC visits	6.6 (1.8)	6.4 (1.8)
Had 8+ ANC visits	53 (33.8)	36(30.0%)
Experienced complications during pregnancy	35 (22.3%)	36 (30.0%)
Delivery experience		
Birth in facility	153 (98.7)	114 (98.3)
Miscarriage	2 (1.27)	4 (3.3)
Month of miscarriage	5 (4.2)	3.8 (2.2)
Baby birth weight (kg)	2.8 (0.8)	3.0 (0.8)
Total amount paid for delivery (USD)	$30.0 (28.5)	$32.1 (49.2)
Paid for delivery using funds from birth plan	67 (43.5%)	73 (62.9%)
Borrowed money or sold things for delivery	72 (45.9%)	42 (35.0%)
Experienced complications during delivery	66 (42.0%)	46 (38.3%)
Cost of travel to facility (USD)	$3.0 (6.0)	$3.3 (5.5)
Length of travel to facility (minutes)	41.9 (37.5)	43.4 (62.6)
Mode of transportation to delivery		
On foot	13 (8.4%)	10 (8.6%)
Bicycle	19 (12.3%)	10 (8.6%)
Motorbike	129 (83.8%)	96 (82.8%)
Other (bus, taxi, car, etc)	25 (15.9%)	17 (14.2%)
Satisfaction With Delivery Experience		
Very Satisfied	149 (96.8%)	113 (97.4%)
Somewhat Satisfied	5 (3.2%)	2 (1.7%)
Very Dissatisfied	0 (0.0%)	1 (0.9%)
Postnatal care experience		
Someone checked on respondent after birth	154 (100.0%)	112 (97.4%)
Someone checked on the baby after birth	154 (100.0%)	116 (100.0%)

Notes: Modes of travel are not mutually exclusive; percentages may sum to more than 100. Cost of transportation & delivery converted using the exchange rate of roughly 7.5 Ghana Cedi to 1 USD in summer 2022.
Abbreviations: ANC = Antenatal Care; kg = kilogram; N=number; USD = United States Dollar

Appendix F: Detailed summary of outcome variables

Table S5. Detailed summary statistics of outcome variables

	Freq (%) or Mean (SD)	Range
Durbar outcomes		
Pregnancy month at first ANC, mean (SD)	3.4 (1.5)	[1,6]
**Initiated ANC in first trimester**	**147 (53.1%)**	---
N days between learning about pregnancy and first ANC visit, mean (SD)	41.4 (27.4)	[0,112]
Decided on her own to come to ANC	95 (34.3%)	---
Accompanied by someone to first ANC	99 (35.7%)	---
Accompanied by husband to first ANC	81 (29.2%)	---
First ANC visit for a checkup	183 (66.1%)	---
Enhanced ANC outcomes		
8+ ANC Visits	89 (32.1%)	---
N ANC Visits,	6.5 (1.8)	[1,11]
N ANC Services, mean (SD)	10.4 (1.4)	[3,11]
Received recommended ANC services	208 (75.1%)	---
N danger signs known, mean (SD)	4.4 (1.9)	[1,9]
Average or above knowledge of danger signs	180 (65.0%)	---
Woman chose ANC frequency	94 (33.9%)	---
Someone accompanied to ANC	131 (47.3%)	---
Husband came to ANC	124 (44.8%)	---
Felt supported by community	191 (70.2%)	---
Felt supported by husband during pregnancy	247 (89.2%)	---
Woman was helped by husband during pregnancy	208 (75.1%)	---
Mother-in-law involved in pregnancy care	177 (64.6%)	---
**Developed birth plan**	**208 (75.1%)**	---
Used birth plan	199 (71.8%)	---
Husband helped with birth plan	180 (65.0%)	---
Paid for delivery using savings	140 (51.1%)	---
Borrowed money or sold possessions to pay for delivery	114 (42.2%)	---
Arranged for blood donor	117 (42.2%)	---
Husband came to delivery	225 (83.3%)	---

Notes: Based on information gathered in Round 1 survey from 277 women who have nonmissing data in both Round 1 and Round 2 surveys. Each cell contains the mean and standard error in parentheses for numeric variables and count and frequency in parentheses for binary variables. The bolded variables are the primary outcome variable to measure the impact of each intervention. Abbreviations: ANC – Antenatal Care; N – number, Freq – Frequency, %– Percent, SD – Standard Deviation.

Appendix G: Regression results in table format

Table S6: Effect of Durbar Intervention

	Pregnancy Month at First ANC	Initiated ANC in first trimester	N days between learning about pregnancy and first ANC visit	Decided on her own to come to ANC	Accompanied by husband to first ANC
Durbar	-0.0	-0.8	0.1	4.4	1.1
95% CI	[-0.4, 0.3]	[-10.4, 12.5]	[-6.5, 4.4]	[-7.1, 12.9]	[-13.5, 19.7]
Observations	277	277	277	277	277
Control Mean	3.4	55.1	40.5	23.9	31.9

Notes: Coefficients represent percentage point change relative to the control mean. 95% confidence intervals in brackets. Control variables include primiparity, age and age squared, education, partner's age and education, religion, wealth index quintile, and catchment fixed effects. Robust standard errors bootstrapped by the wild method. Columns that are grayed out were not specified in the pre-analysis plan. Abbreviations: ANC – antenatal care, CI – confidence interval, N – number.

Table S7: Effect of Enhanced ANC Intervention

	N ANC Visits	8+ ANC Visits	N ANC Services	Received All ANC Services During Pregnancy	N Danger Signs Known	Average or above knowledge of danger signs	Above average knowledge of danger signs	Woman primary decision maker on ANC frequency	Husband accompanied to ANC	Felt supported by Husband during pregnancy	Has Birth Plan	Used Birth Plan	Husband helped with birth plan	Paid for delivery using savings from birth plan	Borrowed money or sold things to pay for delivery	Husband came to delivery
Enhanced ANC	-0.2	-3.1	0.1	5.5	0.5	13.2	11.6	10.1	5.7	0.3	22.1	21.2	28.2	16.4	-7.2	4.0
	[-0.7, 0.3]	[-14.0, 10.5]	[-0.1, 0.2]	[-2.0, 13.6]	[-0.0, 1.0]	[0.1, 24.3]	[-4.5, 26.7]	[-0.5, 18.9]	[-5.3, 14.7]	[-10.2, 10.2]	[9.1, 36.5]	[9.5, 34.3]	[13.0, 42.4]	[0.9, 29.3]	[-18.0, 3.5]	[-5.1, 14.9]
Observations	277	277	277	277	277	277	277	277	277	277	277	277	277	274	270	277
Control Group Mean	6.6	33.8	10.3	71.3	4.1	58.0	38.9	31.2	41.4	87.9	64.3	61.8	51.6	42.9	46.8	77.7

Notes: Coefficients represent percentage point change relative to the control mean. 95% confidence intervals in brackets. Control variables include primiparity, age and age squared, education, partner's age and education, religion, wealth index quintile, and catchment fixed effects. Robust standard errors bootstrapped by the wild method. Columns that are grayed out were not pre-specified. Abbreviations: ANC – antenatal care, CI – confidence interval, N – number.

Appendix H: Robustness Checks – Nonlinear Models

Table S8: Effect of Durbar Intervention - Modified Poisson Regression

	Initiated ANC in first trimester	Decided on her own to come to ANC	Accompanied by husband to first ANC
Durbar	-0.8	4.7	3.0
95% CI	[-14.2, 12.6]	[-18.5, 27.8]	[-16, 21.9]
Observations	277	277	277
Control Mean	-0.8	4.7	3.0

Notes: Binary outcomes estimated using modified Poisson regression models with 95% confidence intervals in brackets. The coefficients represent the average percentage point change from the control mean. Control variables include primiparity, age and age squared, education, partner's age and education, religion, wealth index quintile, occupation, language fixed effects, phone ownership, and insurance. Robust standard errors bootstrapped by the wild method. Regression for ANC in 1st trimester control for month of ANC in prior pregnancy instead of primiparity. Abbreviations: ANC – antenatal care, CI – confidence interval.

Table S9: Effect of Enhanced ANC Intervention - Modified Poisson Regression

Panel A:	Quality of ANC			Support and decision-making during pregnancy			Birth plan and delivery				
	8 + ANC visits	Received recommend ed ANC services	Above average knowledge of danger signs	Woman chose ANC frequency	Husband came to ANC	Felt supported by husband	Developed Birth Plan	Used Birth Plan	Husband helped with birth plan	Paid for delivery using savings	Borrowed money or sold possessions to pay for delivery
Enhanced ANC	-2.7	6.1	10.0	15.1	5.2	0.4	21.4	20.8	27.1	16.4	4.1
95% CI	[-17.5,12.0]	[-15.8,28.1]	[-2.8, 22.7]	[0.9, 29.2]	[-7.3, 17.7]	[-7.4, 8.2]	[11.2, 31.6]	[10.7, 31]	[14.9, 39.3]	[3.2, 29.6]	[-4.5, 12.8]
Observations	277	277	277	277	277	277	277	277	277	274	277
Control Mean	33.8	71.3	38.9	31.2	41.4	87.9	64.3	61.8	51.6	42.9	77.7

Notes: Binary outcomes estimated using modified Poisson regression models with 95% confidence intervals in brackets. The coefficients represent the average percentage point change from the control mean. Control variables include primiparity, age and age squared, education, partner's age and education, religion, wealth index quintile, occupation, phone ownership, and insurance. Robust standard errors bootstrapped by the wild method. Abbreviations: ANC – antenatal care, CI – confidence interval.

Table S10: Effect of Durbar Intervention - Logit Marginal Effects

	Initiated ANC in first trimester	Decided on her own to come to ANC	Accompanied by husband to first ANC
Durbar	-0.9	0.8	1.5
95% CI	[-16.8, 14.9]	[-13.7, 15.3]	[-19.9, 23.0]
Observations	276	260	271
Control Mean	-0.941	0.817	1.544

Notes: Binary outcomes estimated using modified Poisson regression models with 95% confidence intervals in brackets. The coefficients represent the average percentage point change from the control mean. Control variables include primiparity, age and age squared, education, partner's age and education, religion, wealth index quintile, occupation, language fixed effects, phone ownership, and insurance. Robust standard errors bootstrapped by the wild method. Regression for ANC in 1st trimester control for month of ANC in prior pregnancy instead of primiparity. Abbreviations: ANC – antenatal care, CI – confidence interval .

Table S11: Effect of Enhanced ANC Intervention - Logit Marginal Effects

Panel A:	Quality of ANC			Support and decision-making during pregnancy			Birth plan and delivery				
	8 + ANC visits (binary)	Received recommend ed ANC services (binary)	Above average knowledge of danger signs (binary)	Woman chose ANC frequency	Husband came to ANC	Felt supported by husband	Developed Birth Plan	Used Birth Plan	Husband helped with birth plan	Paid for delivery using savings	Borrowed money or sold possessions to pay for delivery
Enhanced ANC	-3.3	6.9	11.1	13.0	5.6	0.7	30.1	21.1	27.0	16.2	-6.5
95% CI	[-13.0,6.3]	[-2.7,16.7]	[-2.3, 24.5]	[-11.1, 37.2]	[-7.9, 19.2]	[-11.5, 13.0]	[15.6, 44.6]	[7.8, 34.4]	[16.9, 37.0]	[2.9, 29.4]	[-19.4,6.4]
Observations	274	174	274	221	274	257	203	274	274	271	267
Control Mean	33.8	71.3	38.9	31.2	41.4	87.9	64.3	61.8	51.6	42.9	46.8

Notes: Binary outcomes estimated using modified Poisson regression models with 95% confidence intervals in brackets. The coefficients represent the average percentage point change from the control mean. Control variables include primiparity, age and age squared, education, partner's age and education, religion, wealth index quintile, occupation, phone ownership, and insurance. Robust standard errors bootstrapped by the wild method. ANC – antenatal care, CI – confidence interval.

Appendix I: Robustness Checks – Leave-one-out Analysis

Table S12: Effect of Durbar Intervention. Excluding Catchment

	8 + ANC visits (binary)	Initiated ANC in first trimester	N days between learning about pregnancy and first ANC visit	Decided on her own to come to ANC	Accompanied by someone to first ANC	Accompanied by husband to first ANC	First ANC visit for a checkup
Excluded Catchment:							
Bolga	0.0	-2.5	0.5	6.3	0.4	1.0	0.5
	[-0.4, 0.3]	[-12.1, 10.1]	[-6.1, 4.8]	[-4.7, 15]	[-15.6, 20.4]	[-13.5, 19.9]	[-10, 9.7]
Garu	-0.1	3.4	-1.3	0.8	4.6	4.3	-3.8
	[-0.5, 0.5]	[-13.3, 18.5]	[-8.5, 4.5]	[-9.6, 10.8]	[-19.3, 26.5]	[-18.5, 25.2]	[-17.4, 10.5]
Namolgo	0.0	-2.5	-1.6	7.7	1.6	0.8	-0.2
	[-0.5, 0.3]	[-12.7, 12.9]	[-8.9, 3]	[-3.8, 14.7]	[-17.3, 25.2]	[-16.2, 23.1]	[-12.6, 9.4]
Sumaduri	0.0	-4.3	1.9	2.3	-4.6	-1.4	7.1
	[-0.5, 0.4]	[-16, 12.8]	[-5, 6.1]	[-14, 12.8]	[-18.4, 13]	[-13.8, 17.6]	[-0.9, 17.1]
Woriyanga	-0.0	2.4	1.3	3.6	1.5	0.4	-5.5
	[-0.5, 0.3]	[-9, 18.8]	[-5.1, 5.4]	[-12.3, 14.5]	[-17.3, 25]	[-16.3, 21.3]	[-20.6, 4.2]

Notes: Coefficients represent percentage point change. 95% confidence intervals in brackets. Control variables include primiparity, age and age squared, education, partner's age and education, religion, wealth index quintile, occupation, phone ownership, and insurance. Standard errors clustered at the village level and bootstrapped by the wild method. Regressions for month of 1st ANC and ANC in 1st trimester control for month of ANC in prior pregnancy instead of primiparity. Abbreviations: ANC – antenatal care, CI – confidence interval, N – number.

Table S13: Effect of Enhanced ANC Intervention. Testing whether excluding catchments from the models changes the results.

	8 + ANC visits	Received recommended ANC services	Above average knowledge of danger signs	Woman primary decision maker on ANC frequency	Husband came to ANC	Felt supported by Husband during pregnancy	Developed Birth Plan	Used Birth Plan	Husband helped with birth plan	Paid for delivery using savings	Husband came to delivery
Excluded Catchment											
Bolga	-0.9	4.7	12.1	10.7	6.2	2.1	20.2	18.9	27.5	15.5**	3.2
	[-10.9, 9.3]	[-1.2, 10.7]	[0.2, 24.1]	[1.6, 19.8]	[-4.3, 16.8]	[-5.2, 9.3]	[11.6, 28.7]	[10.2, 27.7]	[18.3, 36.6]	[4.7, 26.3]	[-4.6, 10.8]
Garu	3.2	2.7	-0.8	5.5	0.9	2.5	27.1	24.7	29.4	6.3	11.0
	[-8.9, 15.6]	[-4.5, 9.8]	[-14.8, 13.4]	[-2.1, 13.1]	[-10.4, 12.3]	[-6.5, 11.8]	[16.3, 37.7]	[13.3, 35.8]	[19, 40]	[-7.7, 20.1]	[2.5, 19.4]
Namolgo	-5.5	7.0	12.8	10.1	5.0	-4.6	16.1	16.4	21.1	17.2***	-0.3
	[-15.3,4.3]	[-0.1,13.9]	[0.5, 25.1]	[-0.4, 20.5]	[-7.2, 17.1]	[-11.6, 2.3]	[7.8, 24.4]	[7.7, 25.2]	[11.4, 30.6]	[5.6, 28.6]	[-6.6, 6]
Sumaduri	-8.0	6.3	21.4	11.9	9.1	-0.4	29.9	27.0	36.4	23.2***	5.7
	[-19.7,3.7]	[-0.9,13.4]	[8.6, 34.2]	[1.4, 22.2]	[-2.2, 20.4]	[-8.3, 7.4]	[19.3, 40.7]	[16, 38.1]	[24.6, 48]	[11.6, 34.7]	[-3.7, 14.9]
Woriyanga	-3.4	7.1	10.1	14.0	5.2	2.5	16.9	18.0	26.1	16.3***	1.9
	[-13.5,6.9]	[0.3,13.8]	[-2, 22.5]	[2.8, 25.2]	[-7, 17.4]	[-6, 11.2]	[9, 24.8]	[9.8, 26.4]	[16.6, 35.5]	[4, 28.2]	[-7.1, 10.7]

Notes: Coefficients represent percentage point change. 95% confidence intervals in brackets. Control variables include primiparity, age and age squared, education, partner's age and education, religion, wealth index quintile, occupation, phone ownership, and insurance. Standard errors clustered at the village level and bootstrapped by the wild method. Regressions for month of 1st ANC and ANC in 1st trimester control for month of ANC in prior pregnancy instead of primiparity.
Abbreviations: ANC – antenatal care, CI – confidence interval.

Appendix J: ANCOVA for Empowerment Outcomes

Table S14: ANCOVA Analysis of Empowerment Outcomes

	Number of empowerment indicators. Max of 9	# of USAID decision-related empowerment indicators. Max of 3	Empowerment Indicator - Work in Last 12 Months	Empowerment Indicator - Method of Payment	Empowerment Indicator - Decisions Over Own Money	Empowerment Indicator - Decisions Over Husband's Money	Empowerment Indicator - Healthcare Decisions	Empowerment Indicator - Major Household Purchases	Empowerment Indicator - Childcare Decisions	Empowerment Indicator - Visiting Decisions	Empowerment Indicator - Cooking Decisions
Enhanced ANC	-0.03	-0.12	4.51	4.90	1.59	2.07	-1.19	-9.03	1.97	-2.60	-5.56
95% CI	[-0.40,0.34]	[-0.33,0.08]	[-0.81,9.84]	[-1.73,11.52]	[-4.70,7.88]	[-7.84,11.98]	[-11.54,9.16]	[-19.80,1.74]	[-7.69,11.63]	[-11.24,6.03]	[-14.16,3.03]
Observations	277	277	277	277	277	277	277	277	277	277	277
Control Mean	4.7	2.0	40.1	31.2	37.6	27.4	61.8	55.4	51.0	82.2	86.6

Notes: Coefficients represent percentage point change. 95% confidence intervals in brackets. Control variables include primiparity, age and age squared, education, partner's age and education, religion, wealth index quintile, and catchment fixed effects. Abbreviations: ANC = antenatal care, CI = confidence interval. USAID = United States Agency for International Development, ANCOVA = Analysis of Covariance.
